# Supplementary material for: Dynamic monitoring of antibody drug conjugates targeting TROP2 or HER2 in breast cancer using circulating tumor cells
Source: Proc Natl Acad Sci U S A. 2026 Jun 17;123(25):e2601563123. doi: 10.1073/pnas.2601563123 (PMC13291658; doi:10.1073/pnas.2601563123)
Supplement: Supplementary file 1 — Appendix 01 (PDF) [file pnas.2601563123.sapp.pdf]

Supplementary Information File

Dynamic Monitoring of Antibody-Drug-Conjugates Targeting TROP2 or HER2 in Breast Cancer using Circulating Tumor Cells.

Avanish Mishra[a,b,#,] Rachel O. Abelman[c,#], Quinn Cunneely[a,b,#], Victor Putaturo[a,b], Akansha A. Deshpande[a,b], Remy Bell[a,b], Elizabeth M. Seider[b], Katherine H. Xu[b], Mythreayi Shan[b], Justin Kelly[b], Shih-Bo Huang[b,e], Olivia Rieur[c], Justine Knape[c], Kaustav A. Gopinathan[a,b], Kruthika Kikkeri[a,b], Jon F. Edd[a,b], John Walsh[a,b], Charles S. Dai[b,c], Leif W. Ellisen[b,c], David T. Ting[b,c], Linda Nieman[b], Mehmet Toner[a,d], Aditya Bardia[c,f] , Daniel A. Haber[b,c,e,*], and Shyamala Maheswaran[b,*]

[a] Center for Engineering in Medicine and Surgery, Massachusetts General Hospital and Harvard Medical School, Charlestown, Massachusetts 02129, USA.

[b] Krantz Family Center for Cancer Research, Massachusetts General Hospital Cancer Center and Harvard Medical School, Charlestown, Massachusetts, 02129, USA.

[c] Division of Hematology Oncology, Massachusetts General Hospital Cancer Center and Department of Medicine, Harvard Medical School, Boston, Massachusetts 02114, USA.

[d] Shriners Children's Hospital, Boston, Massachusetts, 02114, USA.

[e] Howard Hughes Medical Institute, Chevy Chase, Maryland, 20815, USA.

[f] Current Address: Division of Oncology, UCLA Jonsson Cancer Center, Los Angeles, California 90095, USA.

# Denotes equal contribution

*Corresponding authors

Address correspondence to Daniel A. Haber, MD PhD (dhaber@mgh.harvard.edu) and to Shyamala Maheswaran, PhD (smaheswaran@mgh.harvard.edu), both at MGH Cancer Center, Building 149, 13th Street, Charlestown MA 02129

Conflict of Interest Statement

M.T., D.A.H., S.M., and D.T.T. are co-founders of TellBio, a biotechnology company commercializing the CTC-iChip technology. All authors' interests were reviewed and managed by Massachusetts General Hospital and Mass General Brigham in accordance with their conflict-of-interest policies.

Supplementary Table 1: Antibody vendor information

Target	Antibody Name	Vendor	Catalog #	Leica Bond Rx Working Concentration (µg/mL)	Manual Staining Working Concentration (µg/mL)
EpCAM	EpCAM (VU1D9) Mouse mAb (Alexa Fluor® 488 Conjugate)<br>RRID:AB_10692105	Cell Signaling Technology	5198S	1	5
panCK	Pan-Keratin (C11) Mouse mAb (Alexa Fluor® 488 Conjugate)<br>RRID:AB_836889	Cell Signaling Technology	4523S	1	5
CK19	Cytokeratin 19 Monoclonal Antibody (A53-B/A2), Alexa Fluor™ 488<br>RRID:AB_2539532	ThermoFisher Scientific	MA5-18158	1	5
CD16	Alexa Fluor® 647 anti-human CD16 Antibody<br>RRID:AB_492976	BioLegend	302020 (100 tests)	1	5
CD45	Alexa Fluor® 647 anti-human CD45 Antibody<br>RRID:AB_389336	BioLegend	304018 (100 tests)	0.4	5
CD66b	Alexa Fluor® 647 anti-human CD66b Antibody<br>RRID:AB_2563171	BioLegend	305110 (100 tests)	2	5
Trop2	TROP-2 Antibody (001) [DyLight 550]<br>RRID:AB_3435918	Novus Biologicals	NBP2-89492R	Not used	5

Trop2	TROP-2 Antibody (020) [DyLight 550]<br>RRID:AB_3435946	Novus Biologicals	NBP2-89493R	Not used	5
Trop2	Anti-Trop2 antibody [EPR20043]<br>RRID:AB_2811182	abcam	ab214488	5.5	5
HER2	Anti-ErbB2 / HER2 antibody [ICR12]	abcam	ab11710	5	5
Rabbit IgG	Goat Anti-Rabbit IgG H&L (Alexa Fluor® 555)<br>RRID:AB_2722519	abcam	ab150078	4	5
Rat IgG	Goat Anti-Rat IgG H&L (Alexa Fluor® 594)<br>RRID:AB_2756445	abcam	ab150160	4	5

* Pan-Keratin (C11) Mouse mAb (Alexa Fluor® 488 Conjugate) detects endogenous levels of total keratins 4, 5, 6, 8, 10, 13 and 18. The antibody does not cross-react with other keratins.

Supplementary Table 2: Patient cohort

Study ID	Cohort	Lines of Prior Therapy	Target of Prior ADC	Receptor Classification	HER2 IHC	HER2 FISH	Days on Therapy	Baseline CTCs (normalized to 20mL)
MGH001	Primary TROP2-ADC	4	None	ER+/PR+/HER2-	2+	Not Amplified	630	0.0
MGH002	Primary TROP2-ADC	3	None	TNBC	2+	Not Amplified	370	81.5
MGH003	ADC-after-ADC TROP2	5	HER2	ER+/PR+/HER2-	2+	Not Amplified	35	60.0
MGH004	Primary TROP2-ADC	4	None	ER+/PR+/HER2-	0	N/A	357	34.4
MGH005	ADC-after-ADC TROP2	3	HER2	TNBC	1+	N/A	23	6.8
MGH006	Primary TROP2-ADC	3	None	ER+/PR-/HER2-	0	N/A	238	0.0
MGH006	ADC-after-ADC HER2	4	TROP2	ER+/PR-/HER2-	0	N/A	63	293.5
MGH007	Primary TROP2-ADC	5	None	ER+/PR-/HER2-	2+	Not Amplified	450	217.0
MGH007	ADC-after-ADC HER2	6	TROP2	ER+/PR-/HER2-	2+	Not Amplified	42	1055.3
MGH008	Primary TROP2-ADC	3	None	ER+/PR-/HER2-	0	N/A	224	10.3
MGH008	ADC-after-ADC HER2	4	TROP2	ER+/PR-/HER2-	0	N/A	41	0.0
MGH009	Primary TROP2-ADC	0	None	TNBC	1+	N/A	546	4.0
MGH010	Primary TROP2-ADC	1	None	TNBC	1+	N/A	238	0.0
MGH011	Primary TROP2-ADC	6	None	ER+/PR-/HER2-	0	N/A	14	68.8
MGH012	Primary TROP2-ADC	2	None	TNBC	0	N/A	29	6.8
MGH013	Primary TROP2-ADC	0	None	TNBC	0	N/A	28	17.2
MGH014	ADC-after-ADC TROP2	3	HER2	ER+/PR-/HER2-	1+	N/A	91	5.7
MGH016	Primary TROP2-ADC	0	None	TNBC	0	N/A	146	152.3
MGH017	ADC-after-ADC TROP2	9	HER2	ER+/PR+/ HER2-	1+	N/A	111	14.6

MGH018	Primary TROP2-ADC	5	None	ER+/PR+/HER2-	0	N/A	228	0.0
MGH019	ADC-after-ADC TROP2	3	HER2	ER+/PR+/HER2-	2+	Not Amplified	48	428.0
MGH020	Primary HER2-ADC	1	None	ER+/PR+/HER2-	1+	N/A	301	250.0
MGH021	ADC-after-ADC HER2	7	TROP2	ER+/PR-/HER2-	0	N/A	119	15.0
MGH022	Primary HER2-ADC	2	None	ER+/PR+/HER2-	2+	Not Amplified	35	259.5
MGH023	Primary HER2-ADC	3	None	ER+/PR-/HER2-	1+	N/A	301	244.5
MGH024	Primary HER2-ADC	5	None	ER+/PR-/HER2-	2+	Not Amplified	447	13.2
MGH025	Primary HER2-ADC	2	None	ER+/PR-/HER2-	2+	Not Amplified	196	101.3
MGH026	Primary HER2-ADC	2	None	ER+/PR-/HER2-	1+	N/A	42	28.5
MGH027	Primary HER2-ADC	0	None	TNBC	1+	N/A	131	0.0
MGH028	Primary HER2-ADC	2	None	ER-/PR-/HER2+	2+	Amplified	252	14.3
MGH029	ADC-after-ADC HER2	4	TROP2	TNBC	1+	N/A	628	99.5
MGH030	Primary HER2-ADC	0	None	ER+/PR-/HER2-	2+	Not Amplified	419	1480.5
MGH031	Primary HER2-ADC	6	None	ER+/PR+/HER2-	2+	Not Amplified	63	16.9
MGH032	Primary HER2-ADC	1	None	TNBC	2+	Not Amplified	43	2.2
MGH033	Primary HER2-ADC	7	None	ER+/PR+/HER2-	1+	N/A	46	59.5
MGH034	Primary HER2-ADC	6	None	ER+/PR-/HER2-	0	N/A	97	6.5
MGH035	Primary HER2-ADC	2	None	TNBC	1+	N/A	112	2.2
MGH042	Primary HER2-ADC	2	None	ER-/PR-/HER2+	2+	Amplified	364	111.9

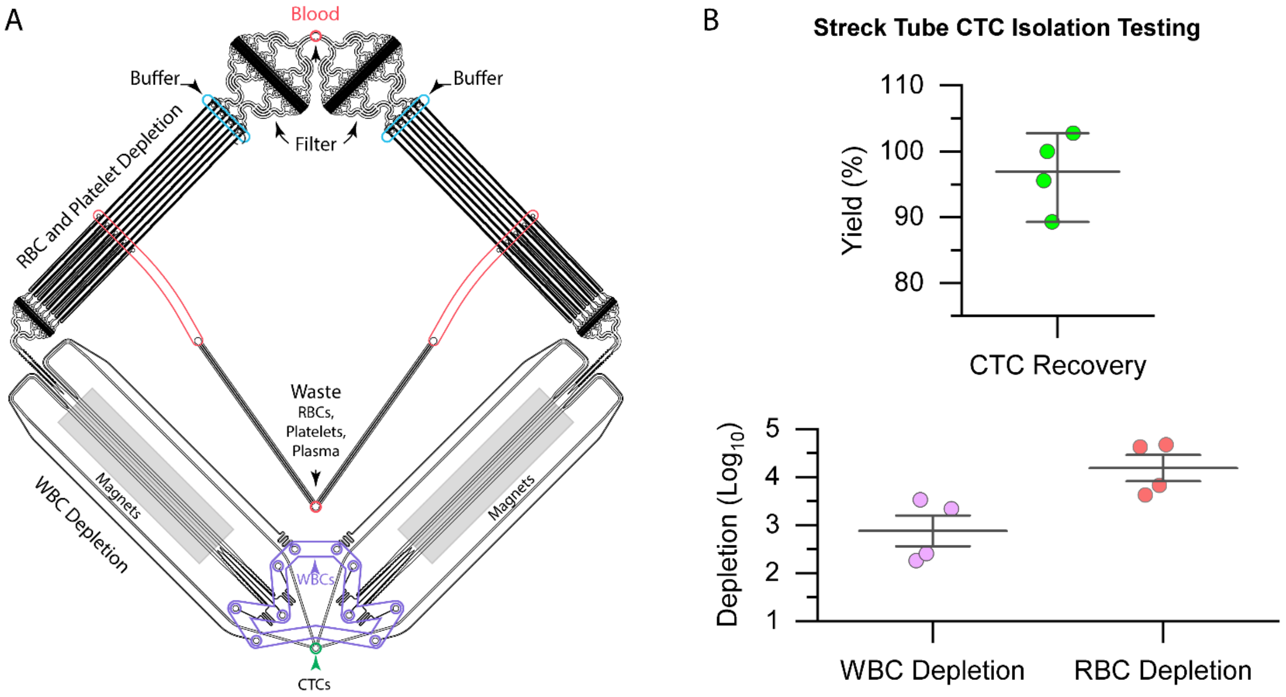

Supplementary Figure 1: **CTC-iChip and cell recovery rates (A)** Schematics of the microfluidic CTC-iChip configuration. **(B)** CTC recovery rates and enrichment levels of fluorescence-tagged CTCs from *in vitro* cultures, spiked into blood from healthy donors, fixed using commercial Streck tubes and stored for 24 hrs before processing through the CTC-iChip, showing 96.9 ± 5.1% CTC recovery, 2.88 ± 0.56 (log_{10}) WBC depletion, and 4.19 ± 0.47 (log_{10}) RBC depletion.

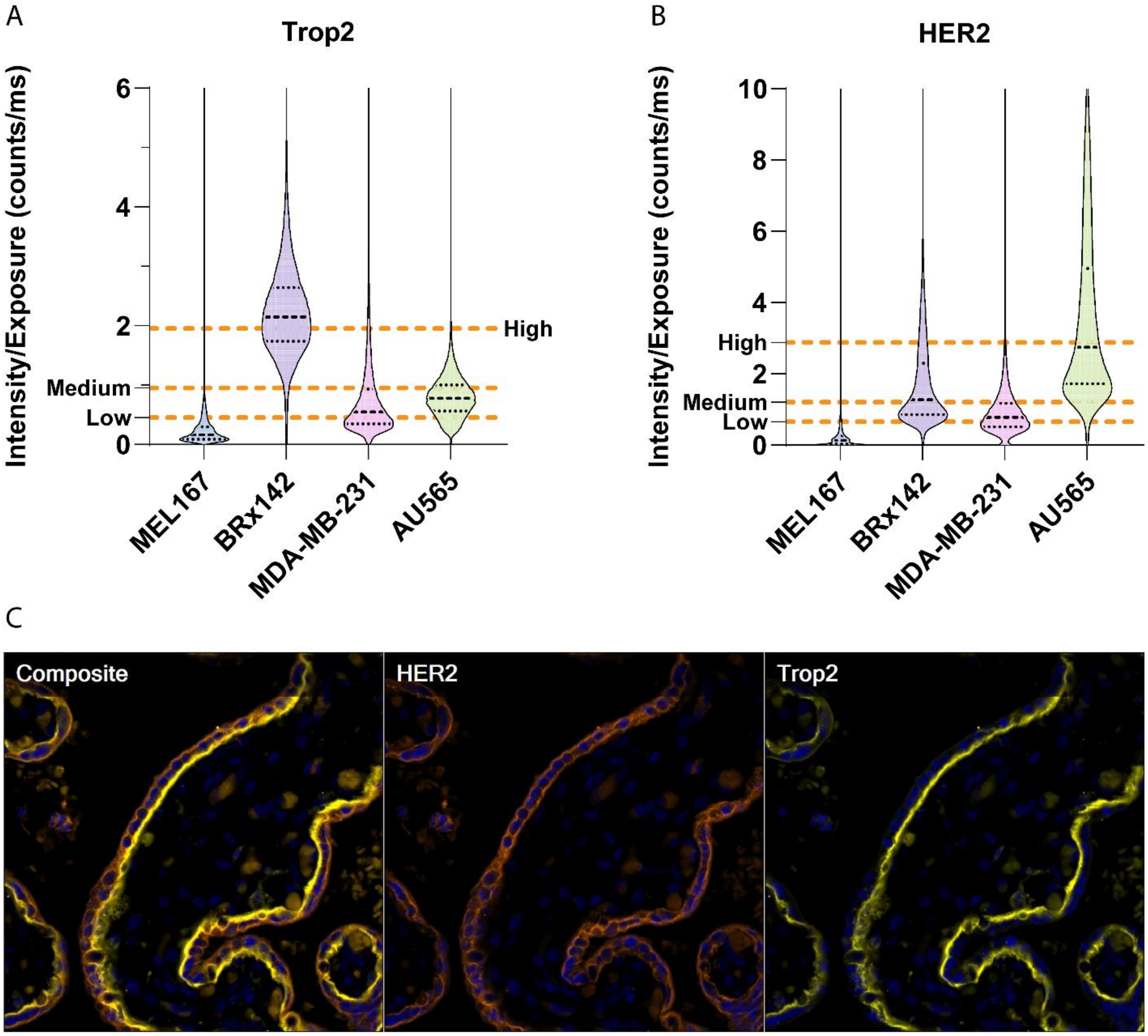

Supplementary Figure 2: **TROP2 and HER2 immunofluorescence staining controls (A-B**) Staining intensity of TROP2 (A) and HER2 (B) proteins in the control cell lines shown, used to establish high, medium, and low expression thresholds (dashed lines) applied to scoring CTCs. **(C)** Validation of TROP2 and HER2 antibodies using placental tissues as positive controls.

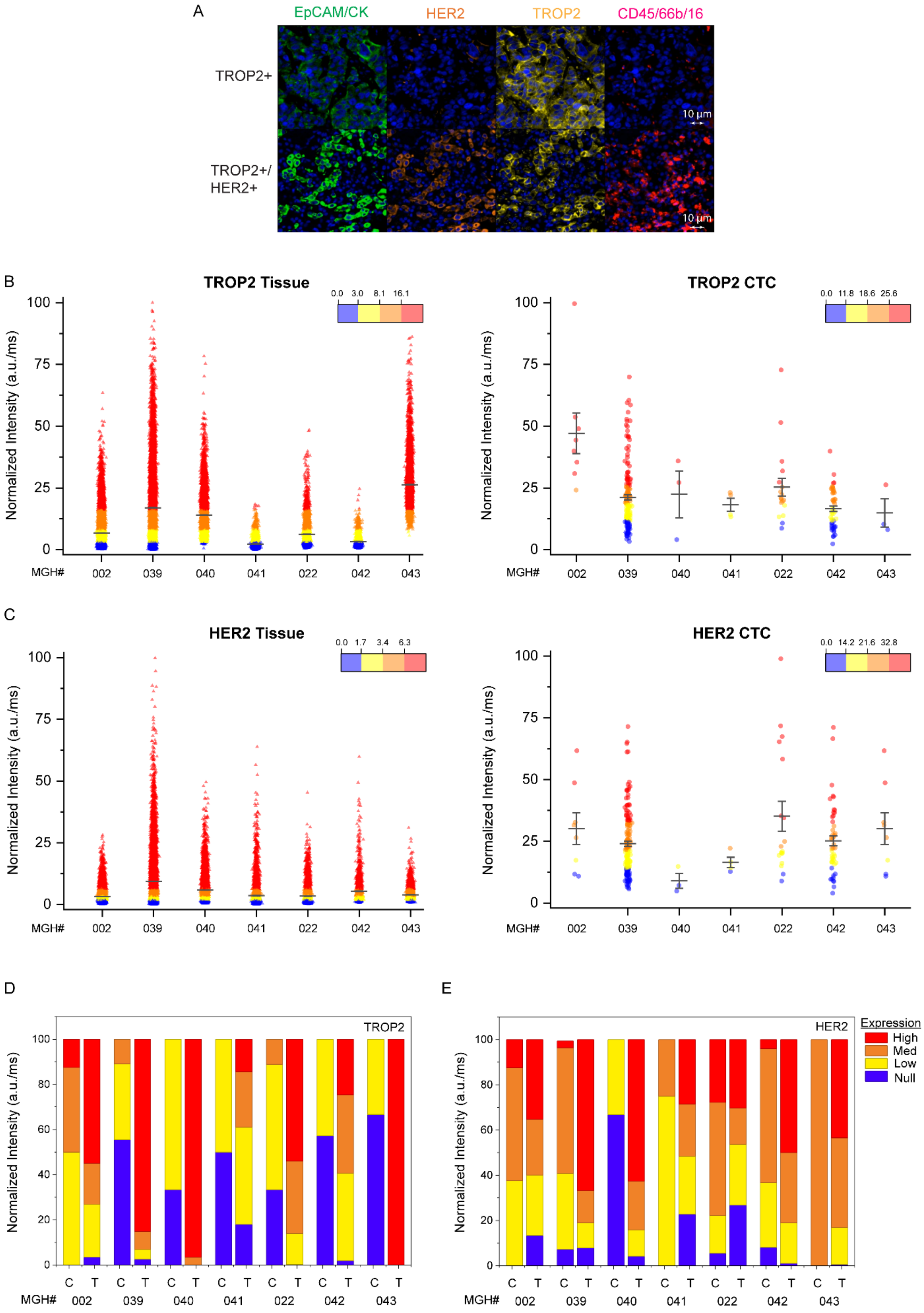

A
EpCAM/CK
HER2
TROP2
CD45/66b/16
TROP2+
TROP2+/ HER2+
10 µm
B
TROP2 Tissue
TROP2 CTC
0.0 3.0 8.1 16.1
0.0 11.8 18.6 25.6
Normalized Intensity (a.u./ms)
MGH#
002
039
040
041
022
042
043
C
HER2 Tissue
HER2 CTC
0.0 1.7 3.4 6.3
0.0 14.2 21.6 32.8
D
E
TROP2
HER2
Expression
High
Med
Low
Null
C
T

Supplementary Figure 3: **Comparison of TROP2 and HER2 expression in CTCs and matched tissue biopsies. (A)** Immunofluorescence images of a metastatic tumor biopsy stained using the nuclear marker DAPI (blue), and the general tumor/epithelial lineage markers (EpCAM, pan-CK, and CK19 (Alexa Fluor 488; green)), as well as TROP2 (Alexa Fluor 555; yellow) and HER2 (Alexa Fluor 594; orange), and leukocyte markers (CD45, CD66b, and CD16 (Alexa Fluor 647; red)). The upper tumor was positive for TROP2 expression only, while the lower tumor was positive for both TROP2 and HER2 expression. **(B)** Single-cell TROP2 expression in metastatic tumor biopsies (tissue) and in CTCs for seven cases in which such matched paired samples were available. For both CTCs and biopsies, single cells are shown as blue (null expression), yellow (low expression), orange (medium expression), or red (high) TROP2 expression, using the quartile classifications shown in Supplementary Figure 2A. The total number of single cells scored are as follows: MGH002: (biopsy: 24,356; CTCs: 8), 039: (biopsy: 14,720; CTCs: 166), 040: (biopsy: 11,704; CTCs: 3), 041: (biopsy: 5,637; CTCs: 4), 022: (biopsy: 5,061; CTCs; 18), 042: (biopsy: 2,163; CTCs: 49), and 043: (biopsy: 5,617; CTCs: 8). **(C)** Analogous comparison of single-cell HER2 expression in tumor biopsies (tissue) and their paired CTCs, as described above. CTCs and tumor cells were classified as null, low, medium, or high in HER2 expression, using the quartile divisions shown in Supplementary Figure 2B. **(D)** Individual comparisons for each of the seven paired clinical samples, showing the fraction and intensity of TROP2 staining for CTCs (C) versus single cells within tumor biopsies (T). **(E)** Analogous comparison of the same seven paired clinical samples for HER2 expression, comparing CTCs and matched biopsy specimens.

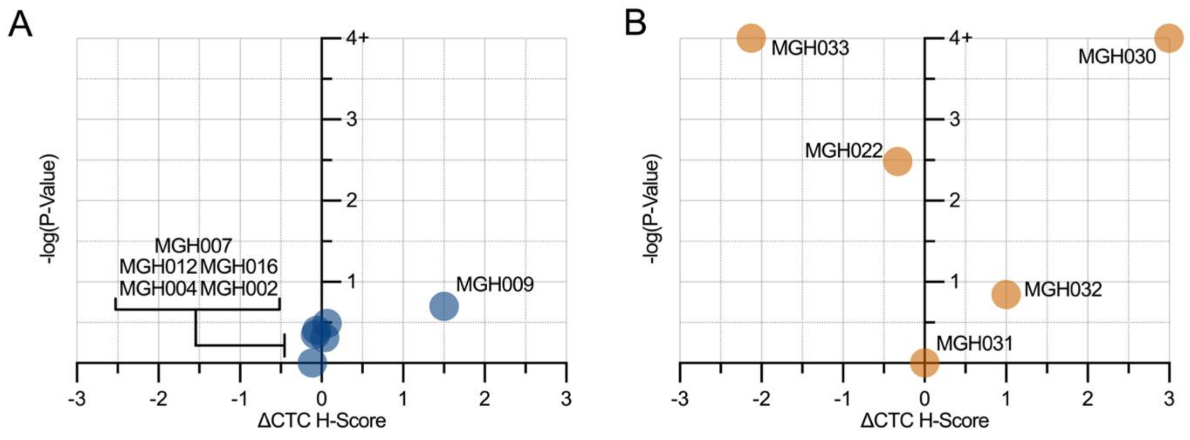

Supplementary Figure 4: Variation in CTC expression patterns at pretreatment baseline versus clinical progression. (A) Plot showing both the change in TROP2 CTC H-score from baseline to progression as well as the log(P-value) (Fisher's exact test) for each of six patients on TROP2-ADC, quantifying the significance of changes in epitope expression between matched pretreatment and ADC-resistant CTCs. H-score is defined as 1×proportion of 'Low' cells + 2×proportion of 'Medium' cells + 3×proportion of 'High' cells. Only one out of five patients (MGH009) demonstrated a meaningful (>1) difference in H-score between draws (increased epitope expression at progression). **(B)** Plot showing the change in HER2 CTC H-score from baseline to progression and the log(P-value) (Fisher's exact test) for each of five patients on HER2-ADC. One patient showed a statistically significant decline in HER2 expression, and one patient showed increased HER2 at progression, with the remaining three patients showing no change in CTC expression.

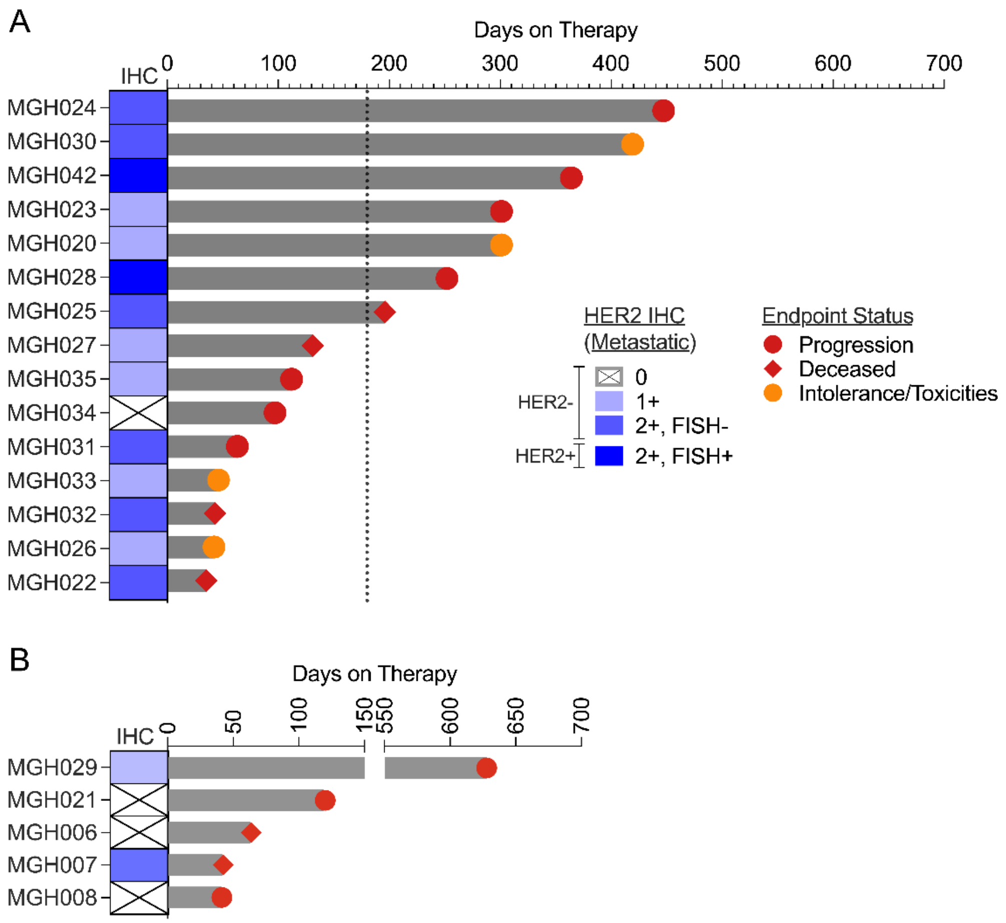

Supplementary Figure 5 Swimmer plot for tumor-based HER2 IHC staining (A) Duration of clinical response (time on therapy) for patients treated with HER2-ADC, as a function of routine clinical staining for HER2 using immunohistochemistry (IHC) and *HER2* gene amplification measurements (FISH). **(B)** Duration of clinical response in patients who had previously received TROP2-ADC prior to treatment with HER2-ADC (ADC-after-ADC cohort), as a function of tumor HER2 staining (clinical IHC staining with FISH assay). Patient MGH029 had only previously received one dose of TROP2-ADC, which was discontinued after six days for toxicity.

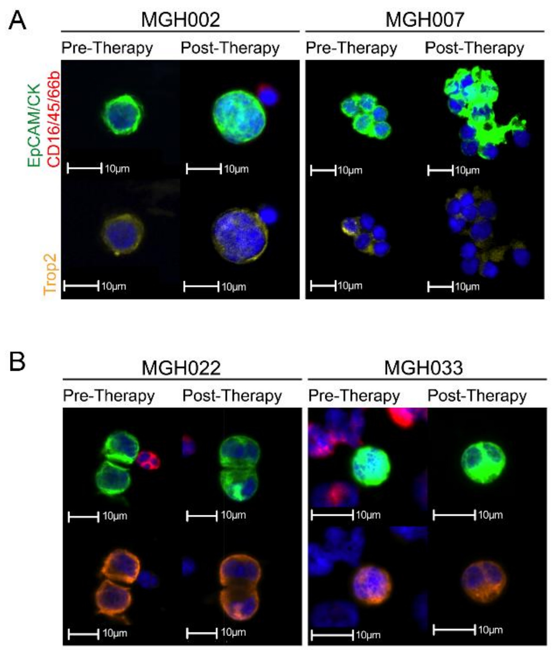

Supplementary Figure 6: **Pattern of epitope staining at clinical progression** (A) Representative images of TROP2 expression in patient-matched CTCs from the time of pre-therapy baseline versus clinical progression, demonstrating unchanged cytological features and epitope staining patterns. (B) Representative images showing similar HER2 expression and CTC morphology in similarly paired pre-therapy and progression samples.
